# Computer tomography-based body surface area evaluation for drug dosage: Quantitative radiology versus anthropomorphic evaluation

**DOI:** 10.1371/journal.pone.0192124

**Published:** 2018-02-14

**Authors:** Antoine Iannessi, Hubert Beaumont, Christophe Hebert, Claire Dittlot, Marie Noëlle Falewee

**Affiliations:** 1 Department of Radiology, Centre Antoine Lacassagne, Nice, France; 2 Research & development, Median Technologies, Valbonne, France; 3 Department of Oncology, Centre Antoine Lacassagne, Nice, France; 4 Department of Gerontology, Centre Hospitalier Universitaire, Nice, France; 5 Department of Nutrition, Centre Antoine Lacassagne, Nice, France; Public Library of Science, UNITED KINGDOM

## Abstract

**Objective:**

The measure of body surface area (BSA) is a standard for planning optimal dosing in oncology. This index is derived from a model having questionable performances. In this study, we proposed measurement of BSA from whole body CT images (iBSA). We tested the reliability of iBSA assessments and simulated the impact of our approach on patient chemotherapy dosage planning.

**Methods:**

We first evaluated accuracy and precision of iBSA in measuring 14 phantom and 11 CT test-retest images.Secondly, we retrospectively analyzed 26 whole body PET-CT scans to evaluate inter-method variability between iBSA and the most used anthropomorphic models, notably the “Du Bois and Du Bois” model. Finally, we simulated the impact on chemotherapy dose planning of capecitabine based on iBSA.

**Results:**

Precision and accuracy of iBSA measurement featured a standard deviation of 1.11% and a mean error of 1.53%. Inter-method variability between iBSA and “Du Bois and Du Bois” assessment featured a standard deviation of 4.11% leading to a reclassification rate of capecitabine of 32.5%.

**Conclusions:**

iBSA could help the oncologist in standardizing assessments for chemotherapy planning. iBSA could also be relevant for applications such as comprehensive body composition and provide a sensitive measurement for changes related to nutritional intake or other metabolism.

## Introduction

The primary objective of dose finding trials for cytotoxic agents in cancer is to assess the safety of an investigational drug. Drug toxicity in an exposed population is correlated with the variability of plasmic concentration [1]. Finding optimal doses of drugs requires a reliable and efficient dose finding design plus a prescription based on a reproducible biomarker. For efficacy trials, this aspect becomes crucial in dosing drugs featuring narrow therapeutic index or for high intensity chemotherapy protocols.

Chemotherapy dosage is currently adjusted based on the body surface area (BSA) [2]. This use is supported by a physiological concept that has not been rigorously demonstrated [3]. Indeed, all pharmacological concepts are based on volume of distribution or clearance without clear correlation with BSA [4]. Yet, BSA-based dosage is currently the standard procedure to estimate limiting drug toxicity during trial phases I and II; this estimation will define subsequent posology in phases III and IV.

Chemotherapy treatment is recurrent during patient follow-up; therefore the reproducibility of assessments is important. Unfortunately, current BSA evaluations are challengeable in terms of reliability of assessment. These procedures are based on simplistic anthropomorphic input data such as weight and height, and are generally derived from a small population sample often not adapted to specific populations of elderly or obese people [5] [6].

Considering that BSA may not be the best approach but is currently the most used parameter to manage drug dosage, it is crucial to improve the reliability of its assessment and to define a straightforward approach for its measure.

Cancer patients almost always undergo CT scans before and during chemotherapy to assess therapeutic response. We hypothesize that these imaging data could also provide valuable anthropomorphic information that is currently unused. In this study, we tested image-based BSA (iBSA) measurements to evaluate the reliability of the assessment and the impact of this approach on patient dosing.

## Materials and methods

### Population data

First, we used phantom data that we acquired at our institutional hospital; then we used test-retest images from the RIDER public dataset [7]; finally, we evaluated whole body CT scan datasets available from our hospital.

Our whole body CT scan (WBCT) dataset consisted of imaging examinations of cancer patients acquired from a Positron Emission Tomography (PET)–CT. By Whole Body PET/CT we mean acquisition from top of the skull to bottom of the feet. In our institution, theses examinations are not usually performed, except for suspicion of lower limb metastasis or melanoma. Over a one year period, from December 2014 to December 2015, we were able to retrospectively collect 35 adults WBCT dataset from the picture archiving system. After a quality control review of the images, 26 WBCT patients were included in the study as 8 out of 35 patient’s images had truncated field of view (arms and legs) and one patient’s image featured motion blur. This study was approved by the monthly Review Board of the institute. External ethic committee’s approval was not asked considering all the aspects of the study. Indeed, patient management information was not part of the study nor modified. Moreover, medical records and imaging data were transmitted and studied retrospectively in a fully anonymized manner.

Acquisitions were performed using a PET-CT scanner (Siemens, BioGraph 40™) using 50% reconstruction overlap and a single slice thickness of 3.0mm with a B30f reconstruction kernel filter. As required for assessing the 18-FDG injection dose, weight and height at the time of the exam were recorded using a scale and a wall mounted stature meter during pre-examination consultation with the physician.

### Quantitative iBSA software/biomarker performances

We evaluated the software performance in computing iBSA as a quantitative imaging biomarker [8].

Based on the standard deviation of inter-method variability of BSA assessments, we extrapolated the variability of drug dosage in performing Monte Carlo simulations. A drug simulation study was based on dose-banding capecitabine monotherapy treating metastatic colorectal cancer or breast cancer [9] [10].

Assessment of iBSA was done using the Lesion Management Solution (LMS) Platform, developed by Median Technologies, Valbonne, France. The image processing technique was fully automatic and required no a priori information.

The processing of CT scans consisted in a first step of segmentation using thresholding to separate patient tissue from air. Then segmentation masks were processed with mathematical morphology and mesh [11] before summing surface of vertexes. Software outputs were iBSA value in cm^2^ and body masks. To ensure the integrity of segmentations, we visually compared them to the original registered CT.

The quantitative measurement of an imaging biomarker is expected to be accurate and reproducible [8].

We validated the software in three different ways; each of these validations relied on a dedicated dataset:

#### Accuracy

We measured the accuracy of surface assessments obtained on CT phantom data of known geometry corresponding to a surface of 0.23m^2^. For this metrological evaluation, we scanned 14 times the same synthetic object acquired at different random positions and locations on the table. Phantom images were processed and the distribution of the percent relative error of the surface area was obtained according to Eq 1.

$$Error=100*(Surf-{Surf}_{Truth})/{Surf}_{Truth}$$ (1)

Where Surf is the surface assessment measured by the software and SurfTruth is the known phantom surface.

#### Precision

We evaluated the precision of surface assessments relying on abdominal thoracic test-retest data. We used 11 test-retest cases from the RIDER database [12]. From the original dataset of 32 patients, we rejected scans acquired with a field of view smaller than body circumference. We performed a Bland Altman analysis to evaluate the precision of surface measurement [13]. Using each couple of test-retest acquisitions, we computed the proportional difference of measurements to assess bias, Standard deviation (SD) and limit of agreement (LoA).

#### Compliance of segmentations

**Fig 1:**
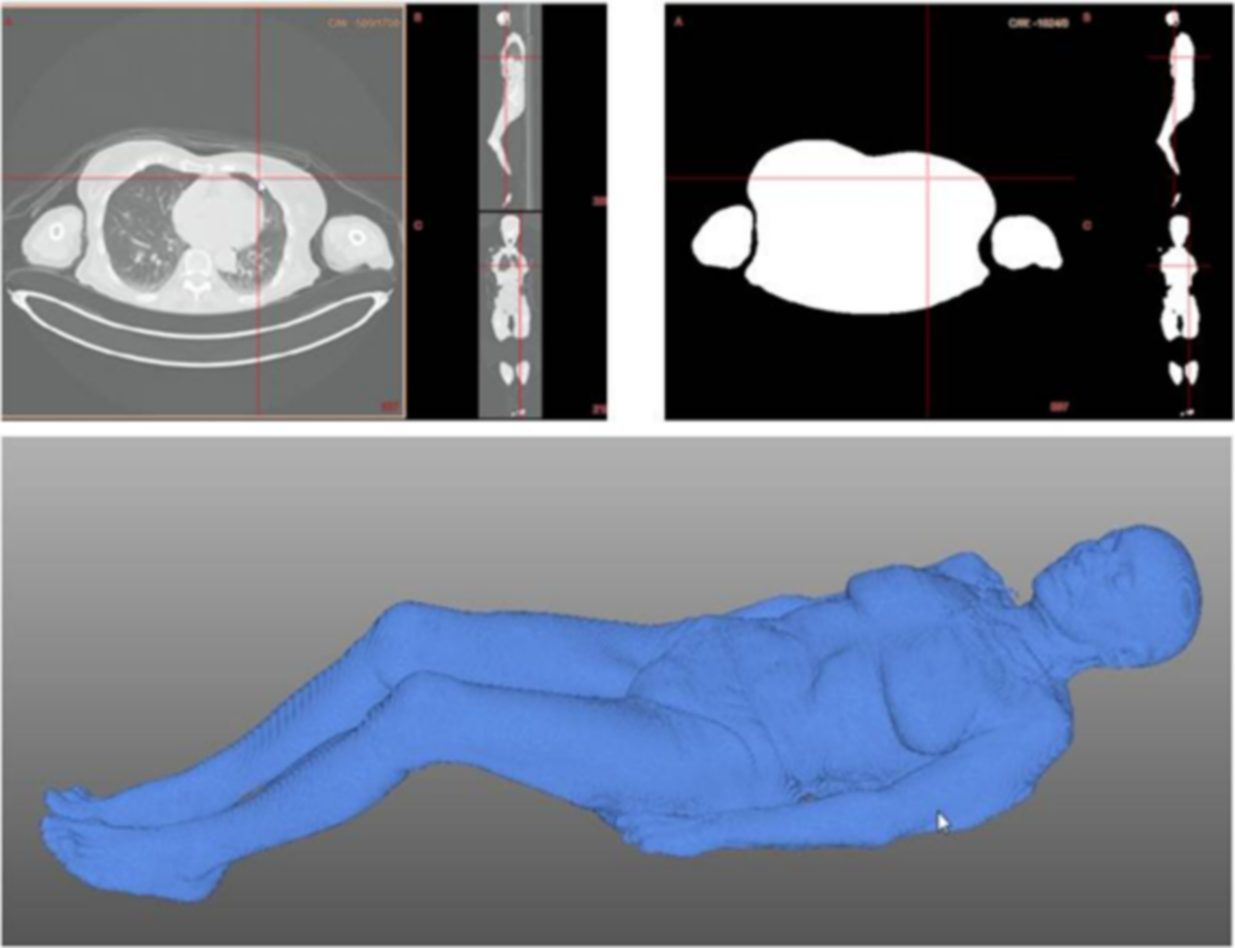
Image-derived BSA quality assessment. Left: Original whole body acquisition, Right: corresponding segmentation mask, Bottom: volume rendering. Two reviewers simultaneously compared original whole body scans, CT scans and segmentation masks to report if corrections were needed.

We checked the correctness of segmentation and, indirectly, confirmed the integrity of measurements, since it is possible to obtain believable measurements with corrupted segmentations. We segmented the whole body data set with the LMS automatic processing tool and asked two radiologists to visually review all segmentations masks. The review included a head to feet side by side comparison of the registered segmentation masks against the corresponding original CT acquisitions. The surface rendering of the segmentations masks was also available (Fig 1). Radiologists were asked to report segmentation quality according to the following rating: “Perfect” when no over- or under-segmentation could be detected, “Good” when limited over- or under-segmentation would be present and “Irrelevant” when segmentation volumes would be over-segmented or with missing regions.

### Inter-method variability

In addition to the software qualification, we were interested in comparing the most used anthropomorphic models of BSA assessments (Du Bois & Du Bois [5], Mosteller [14], Haycock [15], Boyd [16] and Gehan & George [17]) to iBSA by analyzing the inter-method variability significance. We tested 26 patients from the whole body CT scan dataset who had height and weight properly documented. To assess inter-method variability performance, we applied a Bland Altman analysis between anthropomorphic models of BSA, notably the regular Du Bois & Du Bois, and iBSA according to Eq 2:

$$BSA=0.20247*H^{0.725}*W^{0.425}$$ (2)

Bias, SD and LoA were drawn [13] and compared. We compared inter-method variances and bias using respectively F-test and t-test.

### Reclassification rate for capecitabine

To illustrate the impact of the inter-method variability, we simulated the difference between the two BSA assessments in a context of dose banding prescription [18].

We define *re-classification rate* as the number of patients for which dosage has changed when performing assessment either with iBSA or du Bois & du Bois methods. The number of patient for whom dosage has changed band is then divided by the total number of tested patients.

As a final test, we used the estimated inter-method variability to evaluate if this could lead to significant consequences on patient dosage. In order to do this, considering a well-known drug as capecitabine, we simulated the re-classification rate.

**Fig 2:**
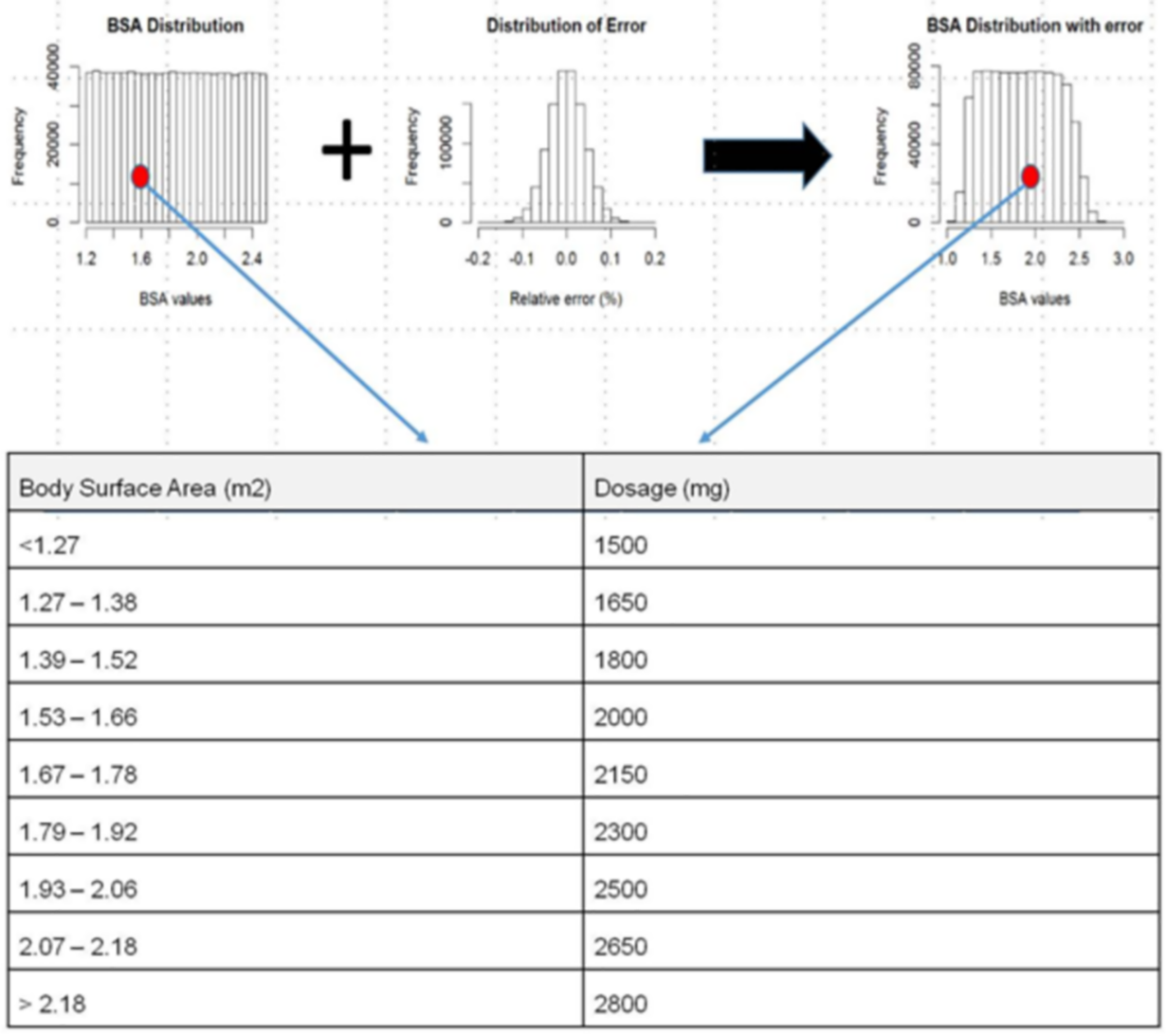
Simulation scheme of re-classification rate calculation according to dose banding adjustment of capecitabine. N = 1000000 BSA samples were uniformly simulated between 1.2 and 2.5m^2^. We assessed the ratio of patient (red circle) of different morphology that would have received a different dosage depending on the method used for BSA assessment. We used a table of standard dose according to a dose-banding protocol.

Using the dosage table displayed on Fig 2, we simulated a first distribution of BSA values to which we added a random percent error. Additive random error was simulated according to inter-method variability we previously analyzed (Fig 2).

Simulations consisted in sampling a uniform distribution of BSA values within 1.2 and 2.5 m^2^ as true reference and the sampling of a Gaussian error distribution N(0,σ) featuring zero means and a SD value evaluated from the Bland Altman analysis of inter-method variation. Thus, simulated assessments (sBSA) were defined as the multiplication of BSA population by the simulated random error as shown in Eq 3:

$${sBSA}_{i}={BSA}_{i}*N(0,\sigma )$$ (3)

Evaluating dosage re-classification rate involved a comparison of patient assignation according to simulated BSA against the sBSA one with respect to dosage bins (Fig 2).

Reclassification rate is computed as indicated on Eq 4.

$$Reclassification\;Rate=1-\frac{\sum_{i=1}^{N}IF[Bins({BSA}_{i})==Bins({sBSA}_{i});1;0]}{N}$$ (4)

All statistical computing was performed using R software (V. 3.1.1). Monte Carlo sampling was computed using the base R package. Confidence interval was computed using the “bootstrap” package.

## Results

### Population data

**Fig 3:**
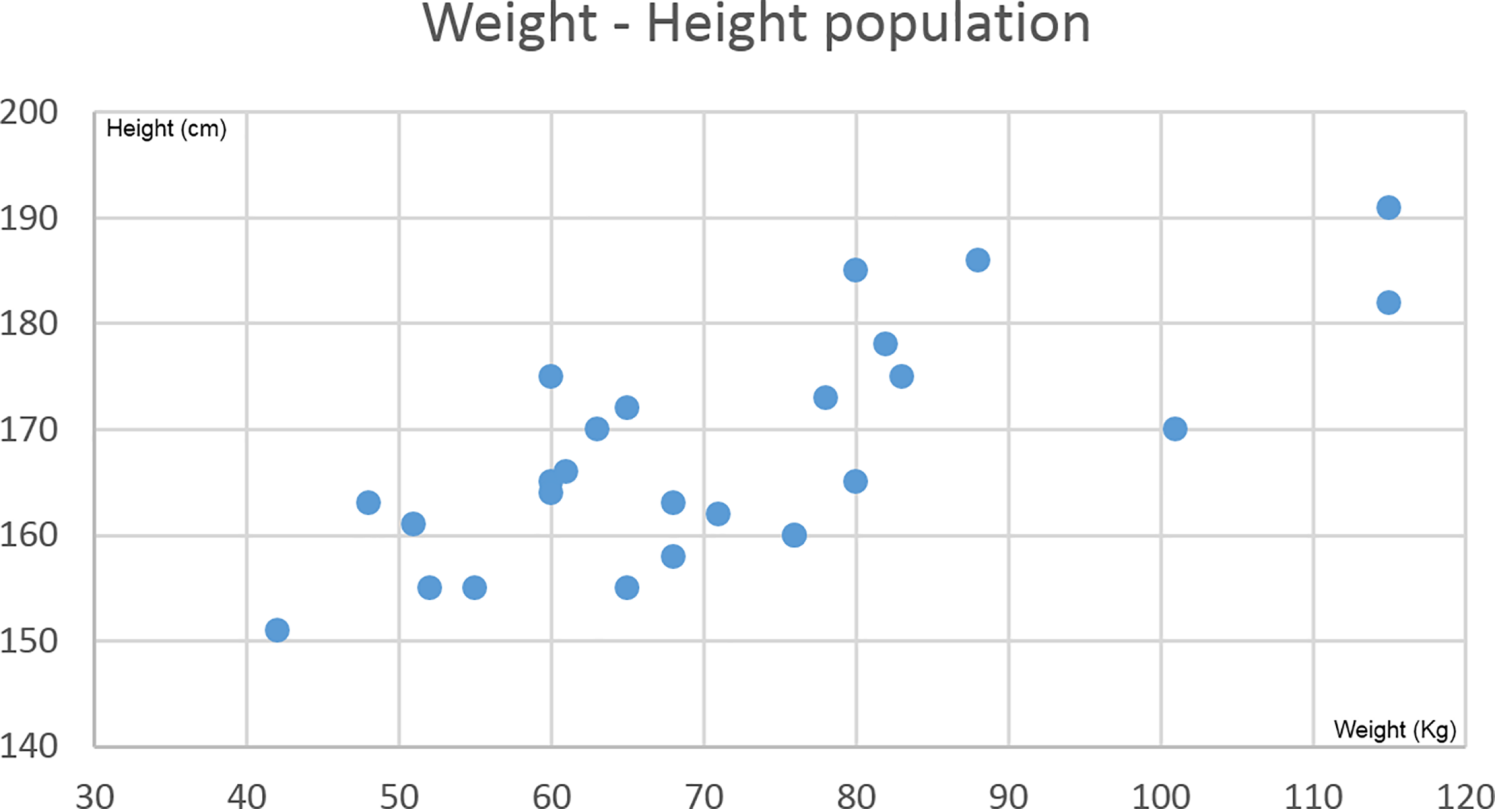
Population of the study. Distribution of weight (horizontal) and height (vertical) of the 26 patients involved in the study.

The population that underwent whole body acquisitions had a weight range of 42–115kg and a height range of 1.51–1.92m (Fig 3). Except for three overweight patients, the studied population was linearly distributed according to a standard weight/height relationship.

50% of this population were males, patients ages ranged from 27 to 91 years old with an average of 66 years.

### Quantitative iBSA software/biomarker performances

**Fig 4:**
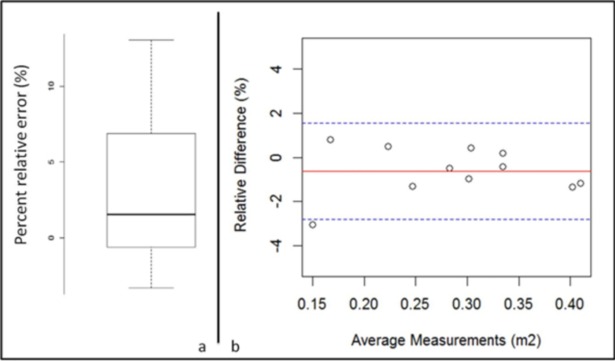
Qualification results of image-derived BSA. a) Accuracy: Box plot of percent relative error in computing BSA of CT phantom. 14 acquisitions of the phantom were repeated with various positions. Median error was 1.54% ranging from -3.3% to 13.06%. b) Precision: Bland Altman plot of test-retest measurement of 11 thoracic and thoracic abdominal patients from the RIDER database.

The accuracy analysis assessed from the percent relative error distribution provided a percent relative error median value of 1.53%, ranging from -3.03% to 13.06% (Fig 4).

Precision was found with a proportional difference standard deviation of 1.11% [0.76%; 2.2%] and a bias of -0.62% [-1.56%; 0.07%] according to the Bland Altman analysis shown in Fig 4.

Subjective visual assessments reported that two reviewers rated 100% of the 35 WBCT segmentations as “Perfect” (We tested the robustness of the segmentation process on the 35 originally selected patients).

### Inter-method variability

In Table 1 Summarized metrics of inter-method variabilities between iBSA and the five anthropomorphic models considered.

**Table 1:** Inter-method comparison between image-derived BSA and main anthropomorphic models. From left to right are reported inter-methods variabilities between iBSA and 1) du Bois & du Bois, 2) Mosteller, 3) Haycock, 4) Boyd and 5) Gehan & George anthropomorphic models. For each model comparison, parameters depicting variability are, from top to bottom: 1) Standard Deviation (StdDev) and 2) Bias.

Anthropomorphic model	Du Bois & Du Bois	Mosteller	Haycock	Boyd	Gehan & George
**StdDev (%)**	4.11	4.49	4.83	4.79	4.55
**Bias (%)**	3.63	2.87	2.33	1.74	2.04

We did not find significantly different variances when comparing iBSA and any of the five other models (iBSA-du Bois & du Bois against iBSA-Haycock (pVal = 0.42)).

We also did not detect significantly different bias when comparing iBSA and any of the four other models (iBSA- du Bois & du Bois against iBSA-Boyd (pVal = 0.13)).

**Fig 5:**
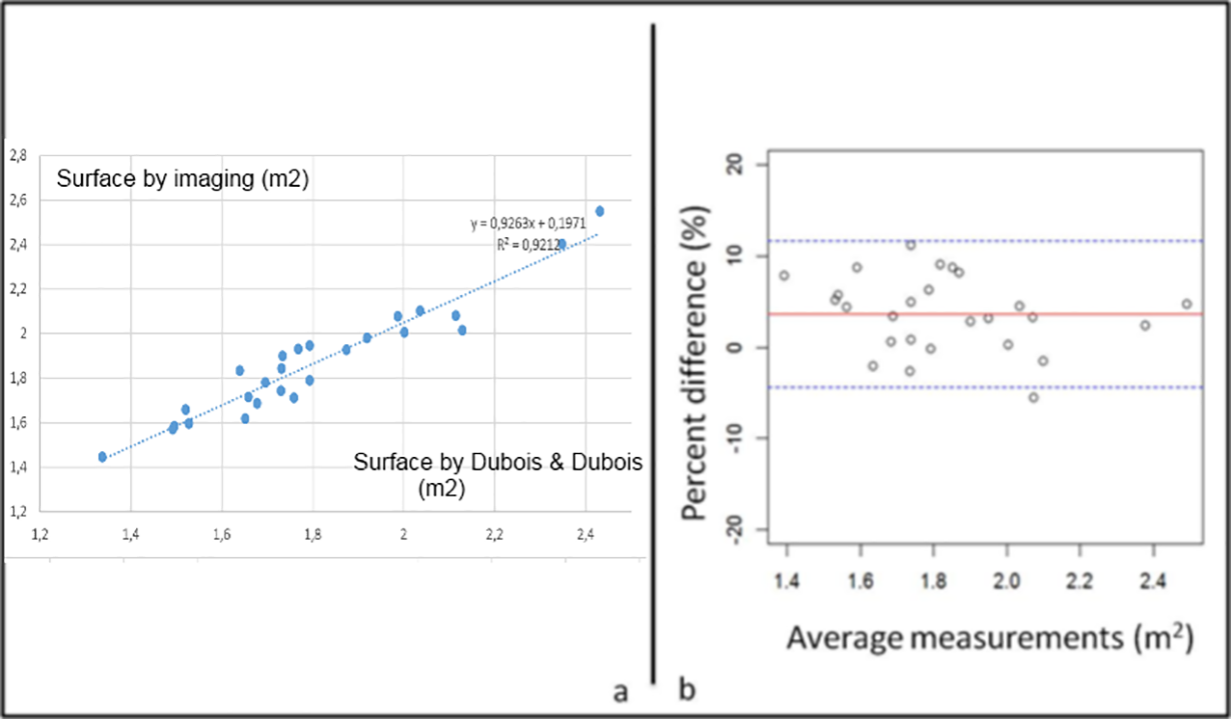
Inter-method comparison between image-derived BSA and Du Bois formula. a) Regression of BSA assessments between du Bois & du Bois method and imaging. Twenty-six patients’ data were considered. b) Bland Altman plot of Surface CT against du Bois & du Bois estimation. The relative difference of error reported a SD = 4.11% with a constant bias of 3.6%.

In particular the inter-method variability between iBSA and du Bois & du Bois featured a standard deviation of 4.11% and a 3.6% bias (Fig 5).

### Reclassification rate for capecitabine

In using the precision of measurement we found, as a parameter in our simulation, the Monte Carlo simulation reported a difference in capecitabine dosage assignment for 32.5% of the sample. 1.1% of the dosages has been shifted from two units.

## Discussion

BSA models that use simple morphological measurements are common procedures that are practical and provide satisfactory performance in most cases; however, several limitations can be reported.

The reliability of assessments computed from equations, such as du Bois and du Bois, directly depends on input reliability as expressed in Eq 5,

$$\frac{\Delta BSA}{BSA}=0.725*\frac{\Delta H}{H}+0.425*\frac{\Delta W}{W}$$ (5)

where an error of 3kg reported by a patient weighing 60kg will make a 5% error on BSA assessment. In the routine, morphological inputs are not always collected in a stringent and standardized way, sometimes even self-reported by patients over the phone.

Second, even if some equations are used more than others, several BSA formulas have been released over several years [19]. Unstandardized practice and use of these formulas across investigational sites can lead to significant inter-site variability.

Finally, several of these formulas have limitations when applied to specific groups, such as sarcopenic or obese patients, using equations that were not designed for these populations or that were derived from significantly under-represented specific populations [5] [6].

By design, iBSA is not prone to these mentioned limitations, iBSA’s reliability is good and it can be routinely available because evaluations are fully automatic. We believe that these features are essential for integration in clinical workflows and use in transversal or longitudinal evaluations.

We recognize a few limitations in computing and analyzing iBSA biomarker.

Our study is based on a dataset of whole body-CT scans that we used to purposely assess iBSA, however patients generally undergo partial-body CT scans, such as thoracic, abdominal, or thoracic-abdominal scans [6]. Thus, extracting the iBSA biomarker for clinical routine would require extrapolating partial measurements to the whole body [20].

Another limitation would arise from the axial field of view for the scanner that may preclude the use of this method in very obese patients whose body contour at least partially exceeds the camera field of view.

Also, the body contact areas, such as the regions between the legs, the arms, and the hands (Fig 1) might have led to body surface measurement errors and correlation coefficient (0.92) vs. du Bois & du Bois—compared to other studies as Villa et al. [21] (r = 0.97).

Even if an assessment bias is generally not regarded as a major limitation, since it can be systematically subtracted from the assessment, we found a software accuracy as low as 1.5%. The test-retest data analysis reported a low random error. The good precision of our measurements should be considered as a worst case situation since our dataset featured a narrow field of view. Therefore, the measured surface was small and the percentage of relative error maximized.

The evaluation of accuracy was obtained using a single non-anthropomorphic phantom because no commercial anthropomorphic phantom is provided with a corresponding BSA feature. We decided to rely on a geometric phantom designed for scanner maintenance and imaged multiple times in all directions. The test-retest dataset was limited (11 patients) for two reasons: 1) these were uncommon data and 2) part of the RIDER data had truncated field of view and were therefore not usable. Furthermore, not all parts of the body were represented in this dataset, only thoracic or thoracic-abdominal scans were included. Our whole-body dataset was reconstructed using 5mm slices unlike images used in the two other validation steps where reconstructions were with 1.25mm thin slices. Involving images obtained with different acquisition parameters at each step of the validation undermined the global analysis of software performances.

As documented in the literature, the volume of distribution and the drug clearance would be the most adapted pharmacokinetic parameters to support a personalized dose adaptation [1]. However, because of the lack of fast and inexpensive biochemical dosing methods, these options are not developed. Moreover, human morphology and BSA are not correlated to body composition and, for example, sarcopenic profiles can be found within a population of obese patients [22].

Among others [23], the most promising perspective of iBSA probably concern drug dosage.

According to our simulation, if iBSA was considered more accurate than BSA, more than 1/3 of the population would have a different dosage of capecitabine. This 150 mg difference represents more than 10% of the dose range between the minimum and the maximum band. Our simulation is performed on common chemotherapy drug with scale-based administration but the narrower the therapeutic index, the more significant this difference is [2].

Practically, iBSA could help scientists define drug dosage more efficiently during dose limiting toxicity finding protocols [24]. Indeed, for clinical research and drug development, the dose adaptation protocol aims at identifying the minimal toxic dose (DMT) for which a preferred therapeutic index is based on the BSA [25]. Even if variability in BSA measures imply variability in DMT estimation, usual protocols tolerate 5% weight uncertainty without dose adaptation but modern chemotherapy requires to maximize dose in order to maximize efficacy. The consequence is the reduction of the therapeutic index that increase the risk of toxicity. De facto, posology is intimately linked to BSA assessment all along patient monitoring. Some at-risk toxicity situations such as a patient losing weight after treatment (head and neck cancer and radiotherapy) or high dose protocols (methotrexate) featuring very narrow therapeutic index require very close surveillance of the prescription and pharmacology [26]. For these situations, it becomes crucial to rely on a sensitive biomarker able to detect BSA changes.

Another area of interest for iBSA is the evaluation of nutritional status. Considering that height is stable, BSA change is correlated with weight change and nutritional status. Having this quantitative information in parallel to follow-up imaging can help in tracking any weight loss due to treatment. In fact, it is clear now that more comprehensive nutritional imaging biomarkers are emerging and they should help physicians to follow patients’ nutritional status and to adapt drug dosage while considering pharmacokinetics [27].

Our study is not suggesting the recommendation of undergoing a whole body CT examination for all patients needing BSA assessment, nevertheless two different applications of iBSA assessment can be considered:

For absolute evaluations based on partial scans, whole body iBSA assessment would require to extrapolate partial iBSA measurements. Another practical option is to normalize the partial iBSA by length z-axis to obtain reproducible nutritional usable information without a whole-body irradiation.

According to the partial derivative equation of du Bois & du Bois (Eq 5), a patient weighting 50kg that is losing 1Kg (2% weight change) will decrease its theoretical BSA value of about 0.8%. In other words, anthropomorphic equations seem to have a limited sensitivity to morphological changes [6]. For patient monitoring, if the precision of iBSA (SD = 1.11%) is confirmed, our approach would improve the detection of BSA change and consequently dose adjustments.

This study provides the first comparison between BSA obtained from routine whole-body CT scans of patients and BSA calculated by an anthropomorphic equation. It shows a remarkably close correlation (R^2^ > 0.9). These data are consistent with literature findings when using 3D laser mapping or post mortem whole-body CT scans [6] [28] [29] [21]. However, several studies argue that DuBois and DuBois' equation is not accurate to predict BSA of overweighed and underweighted individuals. New equations should be developed for children, and for thin or obese patients [21], unlike iBSA evaluations which are not population-dependent.

## Conclusion

We tested BSA evaluations computed from CT scan using a relevant software tool, tested accuracy on a phantom and the precision of the method in several ways checking several potential sources of error. In comparing two evaluation methods of BSA on cytostatic doses, we illustrate the importance of BSA estimations used in current clinical oncology. However, in the field of drug dosage, a novel generation of imaging biomarker could represent the next breakthrough. These imaging biomarkers expected to be more correlated to the pharmacodynamics than BSA.
